# Supplementary material for: Occurrence of cfr-Positive Linezolid-Susceptible Staphylococcus aureus and Non-aureus Staphylococcal Isolates from Pig Farms
Source: Antibiotics (Basel). 2023 Feb 9;12(2):359. doi: 10.3390/antibiotics12020359 (PMC9952267; doi:10.3390/antibiotics12020359)
Supplement: Supplementary file 1 [file antibiotics-12-00359-s001.zip › antibiotics-2198635-supplementary Figures.pdf]

A

GCGAAATGGCTCAATTTTCAGTTGCGAAATACACCCCCACCTTCTATCTCGTCCAGTTGGTTTCCCCAGTCCATAAATATCAGATGAAGACAAGGCACTAAAAGTTTTTATAAAATTGGTTG
ATTTAGGTTTTACATTATGTTGCTAACTTTAATATTTACATTTGAATATTAAAATGTTACAGTACAGGCTAAGTATATTAATTAGTGAAAGGAGGTAATAGCTATGTTAACTGTTTTTAGAATCT
TATTCGGTGAAAATACAAAAATCGGAAACCGTTTAGCTATTAAAGGGAGTAGTCTGTCCTTTTTTAAACTAAACAATGATATAAAAACAGTTGCCGGAGCTTTGCCTCTACTATGGATAAGTT
TAAAACAAGATCATCAACATACTTGTTTTATGTAGTGAAATATGCATATTTGTATGTTTTGACTTTCGGCACCGGTAATTTTTATCTGTGTTTTTTTTGTGTGCAAAAAGAAATTGATTCTTAAC
TAGAGCAAATTGTGAAAGGATGAAAGAAATGAATTTTAATAATAAAACAAAGTATGGTAAAATACAGGAATTTTTAAGAAGTAATAATGAGCCTGATTATAGAATAAAACAAATAACCAATGC
GATTTTTAAACAAAGAATTAGTCGATTTGAGGATATGAAGGTTCTTCCAAAATTACTTAGGGAGGATTTAATAAATAATTTTGGAGAAACAGTTTTGAATATCAAGCTCTTAGCAGAGCAAAA
TTCAGAGCAAGTTACGAAAGTGCTTTTTGAAGTATCAAAGAATGAGAGAGTAGAAACGGTAAACATGAAGTATAAAGCAGGTTGGGAGTCATTTTGTATATCATCACAATGCGGATGTAATT
TTGGGTGTAAATTTTGTGCTACAGGCGACATTGGATTGAAAAAAAAACCTAACTGTAGATGAGATAACAGATCAAGTTTTATACTTCCATTTATTAGGTCATCAAATTGATAGCATTTCTTTTAT
GGGAATGGGTGAAGCTCTAGCCAACCGTCAAGTATTTGATGCTCTTGATTCGTTTACGGATCCTAATTTATTTGCATTAAGTCCTCGTAGACTTTCTATATCAACGATTGGTATTATACCTAG
TATCAAAAAAATAACCCAGGAATATCCTCAAGTAAATCTTACATTTTCATTACACTCACCTTATAGTGAGGAACGCAGCAAATTGATGCCAATAAATGATAGATACCCAATAGATGAGGTAAT
GAATATACTCGATGAACATATAAGATTAACTTCAAGGAAAGTATATATAGCTTATATCATGTTGCCTGGTGTAAATGATTCTCTTGAGCATGCAAACGAAGTTGTTAGCCTTCTTAAAAGTCG
CTATAAATCAGGGAAGTTATATCATGTAAATTTGATACGATACAATCCTACAATAAGTGCACCTGAGATGTATGGAGAAGCAAACGAAGGGCAGGTAGAAGCCTTTTACAAAGTTTTGAAGT
CTGCTGGTATCCATGTCACAATTAGAAGTCAATTTGGGATTGATATTGACGCTGCTTGTGGTCAATTATATGGTAATTATCAAAATAGCCAATAG

B

GCGAAATGGCTCAATTTTCAGTTGCGAAATACACCCCCACCTTCTATCTCGTCCAGTTGGTTTCCCCAGTCCATAAATATCAGATGAAGACAAGGCACTAAAAGTTTTTATAAAATTGGTTG
ATTTAGGTTTTACATTATGTTGCTAACTTTAATATTTACATTTGAATATTAAAATGTTACAGTACAGGCTAAGTATATTAATTAGTGAAAGGAGGTAATAGCTATGTTAACTGTTTTTAGAATCT
TATTCGGTGAAAATACAAAAATCGGAAACCGTTTAGCTATTAAAGGGAGTAGTCTGTCCTTTTTTAAACTAAACAATGATATAAAAACAGTTGCCGGAGCTTTGCCTCTACTATGGATAAGTT
TAAAACAAGATCATCAACATACTTGTTTTATGTAGTGAAATATGCATATTTGTATGTTTTGACTTTCGGCACCGGTAATTTTTATCTGTGTTTTTTTTGTGTGCAAAAAGAAATTGATTCTTAAC
TAGAGCAAATTGTGAAAGGATGAAAGAAATGAATTTTAATAATAAAACAAAGTATGGTAAAATACAGGAATTTTTAAGAAGTAATAATGAGCCTGATTATAGAATAAAACAAATAACCAATGC
GATTTTTAAACAAAGAATTAGTCGATTTGAGGATATGAAGGTTCTTCCAAAATTACTTAGGGAGGATTTAATAAATAATTTTGGAGAAACAGTTTTGAATATCAAGCTCTTAGCAGAGCAAAA
TTCAGAGCAAGTTACGAAAGTGCTTTTTGAAGTATCAAAGAATGAGAGAGTAGAAACGGTAAACATGAAGTATAAAGCAGGTTGGGAGTCATTTTGTATATCATCACAATGCGGATGTAATT
TTGGGTGTAAATTTTGTGCTACAGGCGACATTGGATTGAAAAAAAAACCTAACTGTAGATGAGATAACAGATCAAGTTTTATACTTCCATTTATTAGGTCATAAAATTGATAGCATTTCTTTTAT
GGGAATGGGTGAAGCTCTAGCCAACCGTCAAGTATTTGATGCTCTTGATTCGTTTACGGATCCTAATTTATTTGCATTAAGTCCTCGTAGACTTTCTATATCAACGATTGGTATTATACCTAG
TATCAAAAAAATAACCCAGGAATATCCTCAAGTAAATCTTACATTTTCATTACACTCACCTTATAGTGAGGAACGCAGCAAATTGATGCCAATAAATGATAGATACCCAATAGATGAGGTAAT
GAATATACTCGATGAACATATAAGATTAACTTCAAGGAAAGTATATATAGCTTATATCATGTTGCCTGGTGTAAATGATTCTCTTGAGCATGCAAACGAAGTTGTTAGCCTTCTTAAAAGTCG
CTATAAATCAGGGAAGTTATATCATGTAAATTTGATACGATACAATCCTACAATAAGTGCACCTGAGATGTATGGAGAAGCAAACGAAGGGCAGGTAGAAGCCTTTTACAAAGTTTTGAAGT
CTGCTGGTATCCATGTCACAATTAGAAGTCAATTTGGGATTGATATTGACGCTGCTTGTGGTCAATTATATGGTAATTATCAAAATAGCCAATAG

Figure S1. Sequence analysis of the *cfr* ORF and its promoter region in (A) *cfr*-positive linezolid-resistant and (B) *cfr*-positive linezolid-susceptible strains. Black letters indicate promoter sequences. Red letters with underlines indicate *cfr* ORF sequences. Blue letters indicate a point mutation (C to A) at position 442 in the *cfr* ORF.

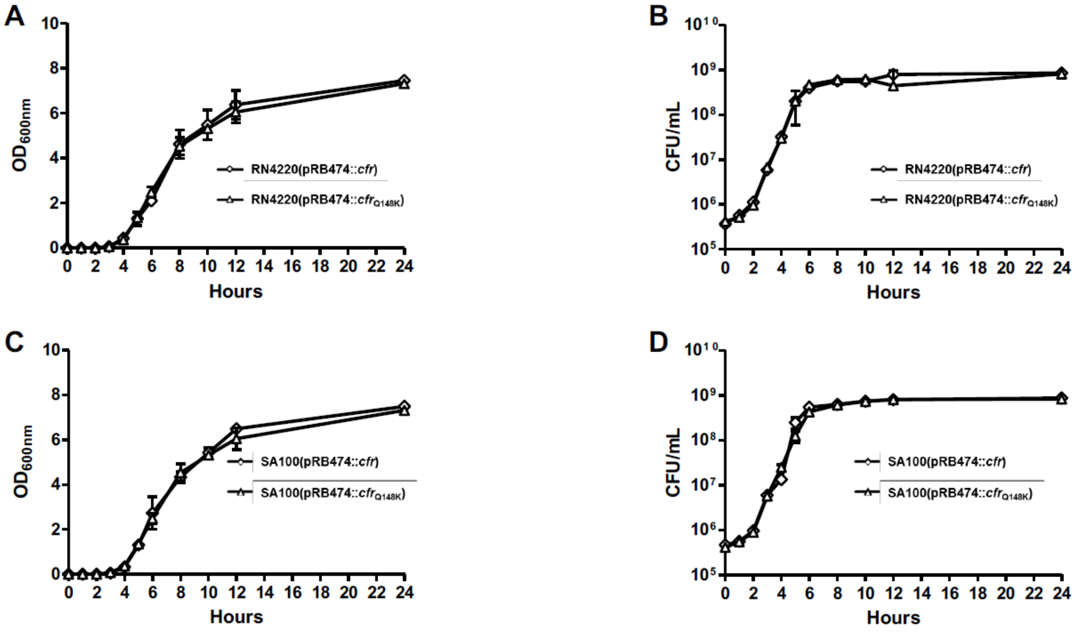

Figure S2. Growth rates of *S. aureus* strains expressing wild-type *cfr* and *cfr*Q148K. Measurement of OD_{600nm} (A and C) and viable cell counts (B and D).
