# Supplementary material for: Occurrence of cfr-Positive Linezolid-Susceptible Staphylococcus aureus and Non-aureus Staphylococcal Isolates from Pig Farms
Source: Antibiotics (Basel). 2023 Feb 9;12(2):359. doi: 10.3390/antibiotics12020359 (PMC9952267; doi:10.3390/antibiotics12020359)
Supplement: Supplementary file 1 [file antibiotics-12-00359-s001.zip › antibiotics-2198635-supplementary tables.pdf]

Table S1. Primer sets for 23S rRNA and ribosomal proteins in staphylococci

	Primer	Sequence (5’-3')
**23S rRNA**	**SA-23S-F**	CGATTTGGGCACTGTCTCA
	**SA-23S-R**	TGTGAACTCTTGGGGGAGAT
	**SE-23S-F**	CCCTAGCTGTGTTGGCTTTC
	**SE-23S-R**	TCGTCATCTTCGAGGGATCT
	**SSC-23S-F**	GTTCCCTCAGAATGGTTGGA
	**SSC-23S-R**	GTGCTTACACCCCGAACCTA
	**SSM-23S-F**	GCATAAGGGAGCTTGACTGC
	**SSM-23S-R**	TCAGCACTTATCCCGTCCAT
**L3**	**SA-L3-F**	AGGAGGTGGACTTTCGATGA
	**SA-L3-R**	TCCTTTTTGCTTCCATGGTT
	**SE-L3-F**	TCTCGTGAACAATTCGAACAA
	**SE-L3-R**	GCCATTATGCAATTTCCTCCT
	**SSC-L3-F**	CGTGAACAATTCGAACAACG
	**SSC-L3-R**	ACCTTGGCGTAATGAAGCAC
	**SSM-L3-F**	CTCGTGAGCAATTCGAACAA
	**SSM-L3-R**	TGCATTTTCCTCCTTTCGTT
**L4**	**SA-L4-F**	CAGGACGTATGGGTGGAAAC
	**SA-L4-R**	CAACTGCCATTTTCACTTGTGT
	**SE-L4-F**	GAAGCGAAAGGAGGAAATTG
	**SE-L4-R**	TCGAAGATTTCTTCAACAGCAA
	**SSC-L4-F**	GGTAACGTGCCAGGTCCTAA
	**SSC-L4-R**	GAAAATTTCTTCGATTGCGATT
	**SSM-L4-F**	AAAGGTAATGTGCCTGGTCCT
	**SSM-L4-R**	TCGCACGTGTATCTACATCAAA
**L22**	**SA-L22-F**	GCTCAAGAAGGAAGCGAAAA
	**SA-L22-R**	CGCATTTAATTTGTTGCGTA
	**SE-L22-F**	CATTGGCCATACTTTTGCAG
	**SE-L22-R**	GCAATATTGATGCGATTTGCT
	**SSC-L22-F**	TGGTCTCGTCGTTCAACAGT
	**SSC-L22-R**	TTTGCTGCACGTTCAATTTC
	**SSM-L22-F**	ACATGCTGCAGACGACAAAA
	**SSM-L22-R**	CCATTTAGCGTCCCAATCAC

Table S2. Comparative analyses of domain V in 23S rRNA and ribosomal proteins

23S rRNA of *S. aureus*

```
ATCC25923      AGTTCCGACCCGCACGAAAGGCGTAACGATTTGGGCACTGTCTCAACGAGAGACTCGGTG
SA2            ************************************************************
SA3            ************************************************************
SA12           ************************************************************
SA16           ************************************************************
SA17           ************************************************************
SA18           ************************************************************
SA19           ************************************************************
SA20           ************************************************************
SA21           ************************************************************

ATCC25923      AAATCATAGTACCTGTGAAGATGCAGGTTACCCGCGACAGGACGGAAAGACCCCGTGGAG
SA2            ************************************************************
SA3            ************************************************************
SA12           ************************************************************
SA16           ************************************************************
SA17           ************************************************************
SA18           ************************************************************
SA19           ************************************************************
SA20           ************************************************************
SA21           ************************************************************

ATCC25923      CTTTACTGTAGCCTGATATTGAAATTCGGCACAGCTTGTACAGGATAGGTAGGAGCCTTT
SA2            ************************************************************
SA3            ************************************************************
SA12           ************************************************************
SA16           ************************************************************
SA17           ************************************************************
SA18           ************************************************************
SA19           ************************************************************
SA20           ************************************************************
SA21           ************************************************************

ATCC25923      GAAACGTGAGCGCTAGCTTACGTGGAGGCGCTGGTGGGATACTACCCTAGCTGTGTTGGC
SA2            ************************************************************
SA3            ************************************************************
SA12           ************************************************************
SA16           ************************************************************
SA17           ************************************************************
SA18           ************************************************************
SA19           ************************************************************
SA20           ************************************************************
SA21           ************************************************************

ATCC25923      TTTCTAACCCGCACCACTTATCGTGGTGGGAGACAGTGTCAGGCGGGCAGTTTGACTGGG
SA2            ************************************************************
SA3            ************************************************************
SA12           ************************************************************
SA16           ************************************************************
SA17           ************************************************************
SA18           ************************************************************
SA19           ************************************************************
SA20           ************************************************************
SA21           ************************************************************

ATCC25923      GCGGTCGCCTCCTAAAAGGTAACGGAGGCGCTCAAAGGTTCCCTCAGAATGGTTGGAAAT
SA2            ************************************************************
SA3            ************************************************************
SA12           ************************************************************
```

```
SA16          ************************************************************
SA17          ************************************************************
SA18          ************************************************************
SA19          ************************************************************
SA20          ************************************************************
SA21          ************************************************************

ATCC25923     CATTCATAGAGTGTAAAGGCATAAGGGAGCTTGACTGCGAGACCTACAAGTCGAGCAGGG
SA2           ************************************************************
SA3           ************************************************************
SA12          ************************************************************
SA16          ************************************************************
SA17          ************************************************************
SA18          ************************************************************
SA19          ************************************************************
SA20          ************************************************************
SA21          ************************************************************

ATCC25923     TCGAAAGACGGACTTAGTGATCCGGTGGTTCCGCATGGAAGGGCCATCGCTCAACGGATA
SA2           ************************************************************
SA3           ************************************************************
SA12          ************************************************************
SA16          ************************************************************
SA17          ************************************************************
SA18          ************************************************************
SA19          ************************************************************
SA20          ************************************************************
SA21          ************************************************************

ATCC25923     AAAGCTACCCCGGGGATAACAGGCTTATCTCCCCCAAGAGTTCACATCGACGGGGAGGTT
SA2           ************************************************************
SA3           ************************************************************
SA12          ************************************************************
SA16          ************************************************************
SA17          ************************************************************
SA18          ************************************************************
SA19          ************************************************************
SA20          ************************************************************
SA21          ************************************************************

ATCC25923     TGGCACCTCGATGTCGGCTCATCGCATCCTGGGGCTG
SA2           *************************************
SA3           *************************************
SA12          *************************************
SA16          *************************************
SA17          *************************************
SA18          *************************************
SA19          *************************************
SA20          *************************************
SA21          *************************************
```

23S rRNA of *S. epidermidis*

```
ATCC12228     CGCTAGCTTACGTGGAGGCGTTGGTGGGATACTACCCTAGCTGTGTTGGCTTTCTAACCC
SE7           ************************************************************
SE9           ************************************************************
SE10          ************************************************************

ATCC12228     GCACCACTTATCGTGGTGGGAGACAGTGTCAGGCGGGCAGTTTGACTGGGGCGGTCGCCT
SE7           ************************************************************
SE9           ************************************************************
SE10          ************************************************************

ATCC12228     CCTAAAAGGTAACGGAGGCGCTCAAAGGTTCCCTCAGAATGGTTGGAAATCATTCATAGA
SE7           ************************************************************
SE9           ************************************************************
SE10          ************************************************************

ATCC12228     GTGTAAAGGCATAAGGGAGCTTGACTGCGAGACCTACAAGTCGAGCAGGGTCGAAAGACG
SE7           ************************************************************
SE9           ************************************************************
SE10          ************************************************************

ATCC12228     GACTTAGTGATCCGGTGGTTCCGCATGGAAGGGCCATCGCTCAACGGATAAAAGCTACCC
SE7           ************************************************************
SE9           ************************************************************
SE10          ************************************************************

ATCC12228     CGGGGATAACAGGCTTATCTCCCCCAAGAGTTCACATCGACGGGGAGGTTTGGCACCTCG
SE7           ************************************************************
SE9           ************************************************************
SE10          ************************************************************

ATCC12228     ATGTCGGCTCATCGCATCCTGGGGCTGTAGTCGGTCCCAAGGGTTGGGCTGTTCGCCCAT
SE7           ************************************************************
SE9           ************************************************************
SE10          ************************************************************

ATCC12228     TAAAGCGGTACGCGAGCTGGGTTCAGAACGTCGTGAGACAGTTCGGTCCCTATCCGTCGT
SE7           ************************************************************
SE9           ************************************************************
SE10          ************************************************************

ATCC12228     GGGCGTAGGAAATTTGAGAGGAGCTGTCCTTAGTACGAGAGGACCGGGATGGACATACCT
SE7           ************************************************************
SE9           ************************************************************
SE10          ************************************************************

ATCC12228     CTGGTGTACCAGTTGTCGTGCCAACGGCATAGCTGGGTAGCTATGTATGGACGGGATAAG
SE7           ************************************************************
SE9           ************************************************************
SE10          ************************************************************

ATCC12228     TGCTGAAAGCATCTAAGCATGAAGCCCCCCTCAAGATGAGATTTCCCAACTTCGGTTATA
SE7           ************************************************************
SE9           ************************************************************
SE10          ************************************************************

ATCC12228     AGATCCCTCGAAGATGACGA
SE7           ********************
SE9           ********************
SE10          ********************
```

23S rRNA of *S. sciuri*

```
NCTC12103     AAGGTTCCCTCAGAATGGTTGGAAATCATTCGCAGAGTGTAAAGGCACAAGGGAGCTTGA
SSC1          ************************************************************
SSC2          ************************************************************

NCTC12103     CTGCGAGACCTACAAGTCGAGCAGGGTCGAAAGACGGACTTAGTGATCCGGTGGTTCCGC
SSC1          ************************************************************
SSC2          ************************************************************

NCTC12103     ATGGAAGGGCCATCGCTCAACGGATAAAAGCTACCCCGGGGATAACAGGCTTATCTCCCC
SSC1          ************************************************************
SSC2          ************************************************************

NCTC12103     CAAGAGTTCACATCGACGGGGAGGTTTGGCACCTCGATGTCGGCTCATCGCATCCTGGGG
SSC1          ************************************************************
SSC2          ************************************************************

NCTC12103     CTGTAGTCGGTCCCAAGGGTTGGGCTGTTCGCCCATTAAAGCGGTACGCGAGCTGGGTTC
SSC1          ************************************************************
SSC2          ************************************************************

NCTC12103     AGAACGTCGTGAGACAGTTCGGTCCCTATCCGTCGTGGGCGTAGGAAATTTGAGAGGAGC
SSC1          ************************************************************
SSC2          ************************************************************

NCTC12103     TGTCCTTAGTACGAGAGGACCGGGATGGACATACCTCTGGTGTACCAGTTGTCGCGCCAG
SSC1          ************************************************************
SSC2          ************************************************************

NCTC12103     CGGCATAGCTGGGTAGCTATGTATGGACGGGATAAGTGCTGAAAGCATCTAAGCATGAAG
SSC1          ************************************************************
SSC2          ************************************************************

NCTC12103     CCCCCCTCAAGATGAGATTTCCCAACTTCGGTTATAAGATCCCTCAAAGATGATGAGGTT
SSC1          ************************************************************
SSC2          ************************************************************

NCTC12103     AATAGGTTCGGGGTGTAAGCAC
SSC1          **********************
SSC2          **********************
```

23S rRNA of *S. simulans*

```
ATCC11046       GCATAAGGGAGCTTGACTGCGAGACCTACAAGTCGAGCAGGGTCGAAAGACGGACTTAGT
SSM1            ************************************************************

ATCC11046       GATCCGGTGGTTCCGCATGGAAGGGCCATCGCTCAACGGATAAAAGCTACCCCGGGGATA
SSM1            ************************************************************

ATCC11046       ACAGGCTTATCTCCCCCAAGAGTTCACATCGACGGGGAGGTTTGGCACCTCGATGTCGGC
SSM1            ************************************************************

ATCC11046       TCATCGCATCCTGGGGCTGTAGTCGGTCCCAAGGGTTGGGCTGTTCGCCCATTAAAGCGG
SSM1            ************************************************************

ATCC11046       TACGCGAGCTGGGTTCAGAACGTCGTGAGACAGTTCGGTCCCTATCCGTCGTGGGCGTAG
SSM1            ************************************************************

ATCC11046       GAAATTTGAGAGGAGCTGTCCTTAGTACGAGAGGACCGGGATGGACATACCTCTGGTGTA
SSM1            ************************************************************

ATCC11046       CCAGTTGTCGTGCCAACGGCATAGCTGGGTAGCTATGTATGGACGGGATAAGTGCTGA
SSM1            **********************************************************
```

L3 of *S. aureus*

```
ATCC25923    AGGAGGTGGACTTTCGATGACCAAAGGAATCTTAGGAAGAAAAATTGGGATGACACAAGT
SA2          ************************************************************
SA3          ************************************************************
SA12         ************************************************************
SA16         ************************************************************
SA17         ************************************************************
SA18         ************************************************************
SA19         ************************************************************
SA20         ************************************************************
SA21         ************************************************************

ATCC25923    ATTCGGAGAAAACGGTGAATTAATCCCTGTAACAGTAGTAGAAGCTAAAGAAAATGTTGT
SA2          ************************************************************
SA3          ************************************************************
SA12         ************************************************************
SA16         ************************************************************
SA17         ************************************************************
SA18         ************************************************************
SA19         ************************************************************
SA20         ************************************************************
SA21         ************************************************************

ATCC25923    ATTACAAAAGAAAACTGTAGAAGTTGATGGATACAACGCAATCCAAGTTGGATTTGAAGA
SA2          ************************************************************
SA3          ************************************************************
SA12         ************************************************************
SA16         ************************************************************
SA17         ************************************************************
SA18         ************************************************************
SA19         ************************************************************
SA20         ************************************************************
SA21         ************************************************************

ATCC25923    CAAAAAAGCATACAAAAAAGATGCAAAATCTAACAAATATGCTAATAAACCAGCTGAAGG
SA2          ************************************************************
SA3          ************************************************************
SA12         ************************************************************
SA16         ************************************************************
SA17         ************************************************************
SA18         ************************************************************
SA19         ************************************************************
SA20         ************************************************************
SA21         ************************************************************

ATCC25923    TCACGCTAAAAAAGCTGACGCAGCACCTAAGCGCTTCATTCGTGAATTCCGCAATGTAGA
SA2          ************************************************************
SA3          ************************************************************
SA12         ************************************************************
SA16         ************************************************************
SA17         ************************************************************
SA18         ************************************************************
SA19         ************************************************************
SA20         ************************************************************
SA21         ************************************************************

ATCC25923    CGTGGATGCTTACGAAGTAGGTCAAGAAGTCTCAGTAGATACTTTTGTAGCTGGCGACGT
SA2          ************************************************************
SA3          ************************************************************
SA12         ************************************************************
SA16         ************************************************************
SA17         ************************************************************
SA18         ************************************************************
SA19         ************************************************************
SA20         ************************************************************
```

```
SA21          ************************************************************

ATCC25923     TATTGACGTAACAGGCGTATCAAAAGGTAAAGGTTTCCAAGGTGCAATTAAACGCCACGG
SA2           ************************************************************
SA3           ************************************************************
SA12          ************************************************************
SA16          ************************************************************
SA17          ************************************************************
SA18          ************************************************************
SA19          ************************************************************
SA20          ************************************************************
SA21          ************************************************************

ATCC25923     ACAATCTCGTGGACCTATGTCACACGGTTCTCATTTCCACAGAGCACCAGGTTCTGTAGG
SA2           ************************************************************
SA3           ************************************************************
SA12          ************************************************************
SA16          ************************************************************
SA17          ************************************************************
SA18          ************************************************************
SA19          ************************************************************
SA20          ************************************************************
SA21          ************************************************************

ATCC25923     TATGGCTTCAGATGCTTCTAGAGTATTCAAAGGCCAAAAAATGCCAGGACGTATGGGTGG
SA2           ************************************************************
SA3           ************************************************************
SA12          ************************************************************
SA16          ************************************************************
SA17          ************************************************************
SA18          ************************************************************
SA19          ************************************************************
SA20          ************************************************************
SA21          ************************************************************

ATCC25923     AAACACTGTAACTGTTCAAAACTTAGAAGTAGTTCAAGTTGACACAGAAAACAAAGTTAT
SA2           ************************************************************
SA3           ************************************************************
SA12          ************************************************************
SA16          ************************************************************
SA17          ************************************************************
SA18          ************************************************************
SA19          ************************************************************
SA20          ************************************************************
SA21          ************************************************************

ATCC25923     CTTAGTAAAAGGTAATGTACCTGGACCTAAAAAAGGTTTAGTAGAAATCAGAACTTCAAT
SA2           ************************************************************
SA3           ************************************************************
SA12          ************************************************************
SA16          ************************************************************
SA17          ************************************************************
SA18          ************************************************************
SA19          ************************************************************
SA20          ************************************************************
SA21          ************************************************************

ATCC25923     TAAAAAAGGTAATAAATAATAAGAAGTGAAAGGAGGAAATGCATAATGGCTAATTATGAT
SA2           ************************************************************
SA3           ************************************************************
SA12          ************************************************************
SA16          ************************************************************
SA17          ************************************************************
SA18          ************************************************************
SA19          ************************************************************
SA20          ************************************************************
SA21          ************************************************************
```

```
ATCC25923      GTTTTAAAATTAGACGGAACTAAATCAGGTTCAATCGAATTAAGCGATGCAGTATTCGGT
SA2            ************************************************************
SA3            ************************************************************
SA12           ************************************************************
SA16           ************************************************************
SA17           ************************************************************
SA18           ************************************************************
SA19           ************************************************************
SA20           ************************************************************
SA21           ************************************************************

ATCC25923      ATTGAGCCAAATAATAGCGTTTTATTCGAAGCTATTAATTTACAACGTGCTTCATTACGT
SA2            ************************************************************
SA3            ************************************************************
SA12           ************************************************************
SA16           ************************************************************
SA17           ************************************************************
SA18           ************************************************************
SA19           ************************************************************
SA20           ************************************************************
SA21           ************************************************************

ATCC25923      CAAGGTACGCATGCTGTTAAGAATCGTTCAGCAGTAAGCGGTGGCGGACGTAAACCATGG
SA2            ************************************************************
SA3            ************************************************************
SA12           ************************************************************
SA16           ************************************************************
SA17           ************************************************************
SA18           ************************************************************
SA19           ************************************************************
SA20           ************************************************************
SA21           ************************************************************

ATCC25923      AAGCAAAAAGGA
SA2            ************
SA3            ************
SA12           ************
SA16           ************
SA17           ************
SA18           ************
SA19           ************
SA20           ************
SA21           ************
```

L3 of *S. epidermidis*

```
ATCC12228     TCTCGTGAACAATTCGAACAACGTACTCATAAACGTTTAATCGACATTGTTAATCCAACA
SE7           ************************************************************
SE9           ************************************************************
SE10          ************************************************************

ATCC12228     CCTAAAACGGTAGATGCTCTAATGGGCTTAAACTTACCATCAGGTGTAGACATCGAAATT
SE7           ************************************************************
SE9           ************************************************************
SE10          ************************************************************

ATCC12228     AAATTATAATAGAACAATATAGGAGGTGGACTTTCGATGACCAAAGGAATCTTAGGAAGA
SE7           ************************************************************
SE9           ************************************************************
SE10          ************************************************************

ATCC12228     AAAATTGGGATGACACAAGTTTTCGGTGAAAATGGTGAATTAATCCCTGTAACAGTTGTA
SE7           ************************************************************
SE9           ************************************************************
SE10          ************************************************************

ATCC12228     GAAGCTAGTCAAAACGTTGTATTACAAAAGAAAACTGAAGAAGTTGATGGTTATAATGCT
SE7           ************************************************************
SE9           ************************************************************
SE10          ************************************************************

ATCC12228     ATCCAAGTAGGTTTTGAAGATAAACAAGCATATAAGAAAGGTTCTAAATCTAATAAATAT
SE7           ************************************************************
SE9           ************************************************************
SE10          ************************************************************

ATCC12228     GCTAATAAACCAGCTGAAGGTCATGCTAAAAAAGCTGACACAGCACCTAAGCGCTTCATT
SE7           ************************************************************
SE9           ************************************************************
SE10          ************************************************************

ATCC12228     CGTGAATTCCGCAACGTTAACGTTGACGAATACGAAGTAGGTCAAGAAGTCTCAGTTGAT
SE7           ************************************************************
SE9           ************************************************************
SE10          ************************************************************

ATCC12228     ACATTCGAAACTGGTGACATCATTGATGTTACAGGCGTTTCAAAAGGTAAAGGTTTCCAA
SE7           ************************************************************
SE9           ************************************************************
SE10          ************************************************************

ATCC12228     GGTGCTATTAAACGTCATGGACAAGGTCGTGGACCAATGGCTCACGGTTCTCATTTCCAT
SE7           ************************************************************
SE9           ************************************************************
SE10          ************************************************************

ATCC12228     AGAGCGCCAGGCTCTGTAGGTATGGCATCAGACGCTTCAAAAGTGTTTAAAGGACAAAAA
SE7           ************************************************************
SE9           ************************************************************
SE10          ************************************************************

ATCC12228     ATGCCAGGACGTATGGGTGGAAACACTGTTACTGTTCAAAACTTAGAAGTCGTTCAAGTT
SE7           ************************************************************
SE9           ************************************************************
SE10          ************************************************************

ATCC12228     GACACTGAAAATAGTGTGATTTTAGTAAAAGGCAATGTACCTGGACCTAAAAAAGGTTTA
SE7           ************************************************************
SE9           ************************************************************
SE10          ************************************************************
```

```
ATCC12228      GTAGAAATCACAACTTCAATAAAAAAAGGTAATAAATAATTAAGAAGCGAAAGGAGGAAA
SE7            ************************************************************
SE9            ************************************************************
SE10           ************************************************************

ATCC12228      TTGCATAATGGC
SE7            ************
SE9            ************
SE10           ************
```

L3 of *S. sciuri*

```
NCTC12103     CGTGAACAATTCGAACAACGTACGCATAAACGTTTAATCGACATTCTTAACCCTACACCA
SSC1          ************************************************************
SSC2          ************************************************************

NCTC12103     AAAACAGTTGATGCTCTTATGGGCTTAAACTTACCATCAGGTGTAGACATCGAAATCAAA
SSC1          ************************************************************
SSC2          ************************************************************

NCTC12103     TTATAATTCATTAAAATAACAGGAGGTGCGACTTTCGATGACCAAAGGAATCTTAGGAAG
SSC1          ************************************************************
SSC2          ************************************************************

NCTC12103     AAAAATTGGAATGACACAAGTATTCGCAGAAAACGGTGAATTAATACCAGTTACTGTAGT
SSC1          ************************************************************
SSC2          ************************************************************

NCTC12103     AGAAGCTAGTCAAAACGTAGTATTACAAACTAAAACTGAAGAAGTGGACGGCTACAACGC
SSC1          ************************************************************
SSC2          ************************************************************

NCTC12103     TGTTCAAATCGGTTTTGAAGACAAACAAGCATATAAAAAAGATCGTAAATCTAACAAATA
SSC1          ************************************************************
SSC2          ************************************************************

NCTC12103     TGCTACTAAAGCTGCTGAAGGCCATGCTAAAAAAGCAGGTACAGCACCTAAGCGCTTCAC
SSC1          ************************************************************
SSC2          ************************************************************

NCTC12103     TCGTGAATTCAGAAACATTGATGTTTCAGCATACGAGGTAGGTCAAGAAGTCACTGTAGA
SSC1          ************************************************************
SSC2          ************************************************************

NCTC12103     TACTTTCCAAGCAGGAGACATTATTGATGCAACAGGCGTTTCAAAAGGTAAAGGTTTCCA
SSC1          ************************************************************
SSC2          ************************************************************

NCTC12103     AGGCTCAATTAAACGTCACGGATTCTCTCGTGGACCAATGAGTCACGGTTCTCGTTACCA
SSC1          ************************************************************
SSC2          ************************************************************

NCTC12103     CAGAGGCTCTGGTTCAATGGGTATGGCTTCAGATGCTTCTAAAGTATTTAAAGGTAAAGA
SSC1          ************************************************************
SSC2          ************************************************************

NCTC12103     ATTACCAGGCCGTATGGGCGGAAACACAGTTACTATGCAAAACTTAGAAGTAGTTAAAGT
SSC1          ************************************************************
SSC2          ************************************************************

NCTC12103     TGATGCAGAAAATAACGTAATTTTAGTTAAAGGTAACGTGCCAGGTCCTAAAAAAGGTTT
SSC1          ************************************************************
SSC2          ************************************************************

NCTC12103     AGTAAAACTTACAACTTCAATTAAAAAAGGTAATAAATAATTATTTAGGTGAAAGGAGGA
SSC1          ************************************************************
SSC2          ************************************************************

NCTC12103     AACAACATTATGGCTAATATTGATGTATTAAAAGTAGATGGATCAAAATCAGGTTCTATC
SSC1          ************************************************************
SSC2          ************************************************************

NCTC12103     GAATTAAATGATAGTGTATTTGGTATCGAACCAAACCAACACGTATTATTCGAAGCAGTC
SSC1          ************************************************************
SSC2          ************************************************************
```

NCTC12103	AACTTACAACGTGCTTCATTACGCCAAGGT
SSC1	******************************
SSC2	******************************

L3 of *S. simulans*

```
ATCC11046      CTCGTGAGCAATTCGAACAACGTACTCATAAACGTTTAATCGACATTGTTAACCCTACAC
SSM1           ************************************************************

ATCC11046      CAAAAACTGTTGATGCTCTTATGGGCTTAAACTTACCATCAGGTGTAGACATCGAAATTA
SSM1           ************************************************************

ATCC11046      AATTATAATAGAAAATCATAGGAGGTGGACTTTCGATGACCAAAGGAATCTTAGGAAGAA
SSM1           ************************************************************

ATCC11046      AAATCGGGATGACACAAGTATTCGGTGAAAACGGAGACTTAATCCCAGTAACTGTAGTAG
SSM1           ************************************************************

ATCC11046      AAGCAGGACAAAACGTAGTATTACAAAAGAAAACTGAAGAGGTAGACGGTTACAACGCTA
SSM1           ************************************************************

ATCC11046      TCCAAGTAGGTTTTGAAGACAAACAAGCATACAAAAAAGACAGCAAGTCAAATAAATATG
SSM1           ************************************************************

ATCC11046      CTAATAAACCAGCTGAAGGTCACGCTAAAAAAGCTGACACAGCTCCTAAGCGCTTCATTC
SSM1           ************************************************************

ATCC11046      GTGAATTCAGAAACATCAATGTTGATGAATACGAAGTAGGTCAAGAAGTCTCAGTTGATA
SSM1           ************************************************************

ATCC11046      CATTTGAAGCTGGGGACATCATTGACGTAACTGGAGTATCTAAAGGTAAAGGTTTCCAAG
SSM1           ************************************************************

ATCC11046      GCAATATCAAACGTCATGGCCACGGTCGTGGACCAATGTCTCACGGTTCTCATTTCCACA
SSM1           ************************************************************

ATCC11046      GAGCGCCAGGTTCTGTAGGTATGGCATCTGACGCATCTAAAGTATTTAAAGGTCACAAAA
SSM1           ************************************************************

ATCC11046      TGCCAGGTCGTATGGGTGGCAACACAGTGACAGTACAAAACTTAGAAGTTGTACAAATCG
SSM1           ************************************************************

ATCC11046      ATACAGAAAACAACGTTATCTTAGTTAAAGGTAATGTGCCTGGTCCTAAAAAAGGATTAG
SSM1           ************************************************************

ATCC11046      TAGAAATCAAAACTTCAATTAAAAAAGGTAACAAATAATTAATTAACGAAAGGAGGAAAA
SSM1           ************************************************************

ATCC11046      TGCA
SSM1           ****
```

L4 of *S. aureus*

```
ATCC25923       CAGGACGTATGGGTGGAAACACTGTAACTGTTCAAAACTTAGAAGTAGTTCAAGTTGACA
SA2             ************************************************************
SA3             ************************************************************
SA12            ************************************************************
SA16            ************************************************************
SA17            ************************************************************
SA18            ************************************************************
SA19            ************************************************************
SA20            ************************************************************
SA21            ************************************************************

ATCC25923       CAGAAAACAAAGTTATCTTAGTAAAAGGTAATGTACCTGGACCTAAAAAAGGTTTAGTAG
SA2             ************************************************************
SA3             ************************************************************
SA12            ************************************************************
SA16            ************************************************************
SA17            ************************************************************
SA18            ************************************************************
SA19            ************************************************************
SA20            ************************************************************
SA21            ************************************************************

ATCC25923       AAATCAGAACTTCAATTAAAAAAGGTAATAAATAATAAGAAGTGAAAGGAGGAAATGCAT
SA2             ************************************************************
SA3             ************************************************************
SA12            ************************************************************
SA16            ************************************************************
SA17            ************************************************************
SA18            ************************************************************
SA19            ************************************************************
SA20            ************************************************************
SA21            ************************************************************

ATCC25923       AATGGCTAATTATGATGTTTTAAAATTAGACGGAACTAAATCAGGTTCAATCGAATTAAG
SA2             ************************************************************
SA3             ************************************************************
SA12            ************************************************************
SA16            ************************************************************
SA17            ************************************************************
SA18            ************************************************************
SA19            ************************************************************
SA20            ************************************************************
SA21            ************************************************************

ATCC25923       CGATGCAGTATTCGGTATTGAGCCAAATAATAGCGTTTTATTCGAAGCTATTAATTTACA
SA2             ************************************************************
SA3             ************************************************************
SA12            ************************************************************
SA16            ************************************************************
SA17            ************************************************************
SA18            ************************************************************
SA19            ************************************************************
SA20            ************************************************************
SA21            ************************************************************

ATCC25923       ACGTGCTTCATTACGTCAAGGTACGCATGCTGTTAAGAATCGTTCAGCAGTAAGCGGTGG
SA2             ************************************************************
SA3             ************************************************************
SA12            ************************************************************
SA16            ************************************************************
SA17            ************************************************************
SA18            ************************************************************
SA19            ************************************************************
SA20            ************************************************************
```

```
SA21          ************************************************************

ATCC25923     CGGACGTAAACCATGGAAGCAAAAAGGAACAGGTCGTGCTCGTCAAGGTACAATCCGTGC
SA2           ************************************************************
SA3           ************************************************************
SA12          ************************************************************
SA16          ************************************************************
SA17          ************************************************************
SA18          ************************************************************
SA19          ************************************************************
SA20          ************************************************************
SA21          ************************************************************

ATCC25923     TCCACAATGGCGTGGCGGTGGTATCGTATTCGGACCAACTCCAAGAAGTTATGCATACAA
SA2           ************************************************************
SA3           ************************************************************
SA12          ************************************************************
SA16          ************************************************************
SA17          ************************************************************
SA18          ************************************************************
SA19          ************************************************************
SA20          ************************************************************
SA21          ************************************************************

ATCC25923     AATGCCTAAGAAAATGCGTCGTTTAGCTTTACGCTCAGCATTATCTTTCAAAGTTCAAGA
SA2           ************************************************************
SA3           ************************************************************
SA12          ************************************************************
SA16          ************************************************************
SA17          ************************************************************
SA18          ************************************************************
SA19          ************************************************************
SA20          ************************************************************
SA21          ************************************************************

ATCC25923     GAATGGCTTAACTGTAGTTGACGCATTCAACTTCGAAGCTCCAAAAACTAAAGAATTCAA
SA2           ************************************************************
SA3           ************************************************************
SA12          ************************************************************
SA16          ************************************************************
SA17          ************************************************************
SA18          ************************************************************
SA19          ************************************************************
SA20          ************************************************************
SA21          ************************************************************

ATCC25923     AAATGTATTATCTACATTAGAACAACCTAAAAAAGTATTAGTAGTTACTGAAAACGAAGA
SA2           ************************************************************
SA3           ************************************************************
SA12          ************************************************************
SA16          ************************************************************
SA17          ************************************************************
SA18          ************************************************************
SA19          ************************************************************
SA20          ************************************************************
SA21          ************************************************************

ATCC25923     TGTAAATGTTGAATTATCAGCACGCAACATCCCTGGCGTTCAAGTGACAACTGCTCAAGG
SA2           ************************************************************
SA3           ************************************************************
SA12          ************************************************************
SA16          ************************************************************
SA17          ************************************************************
SA18          ************************************************************
SA19          ************************************************************
SA20          ************************************************************
SA21          ************************************************************
```

```
ATCC25923    TTTAAATGTTTTAGATATCACTAATGCTGACAGCTTAGTAATTACTGAAGCTGCTGCTAA
SA2          ************************************************************
SA3          ************************************************************
SA12         ************************************************************
SA16         ************************************************************
SA17         ************************************************************
SA18         ************************************************************
SA19         ************************************************************
SA20         ************************************************************
SA21         ************************************************************

ATCC25923    AAAAGTTGAGGAGGTGCTCGGATAATGGAAGCAAGAGATATTCTTAAGCGCCCCGTAATC
SA2          ************************************************************
SA3          ************************************************************
SA12         ************************************************************
SA16         ************************************************************
SA17         ************************************************************
SA18         ************************************************************
SA19         ************************************************************
SA20         ************************************************************
SA21         ************************************************************

ATCC25923    ACTGAGAAATCTTCTGAAGCAATGGCTGAAGACAAATACACTTTCGACGTTGATACTCGT
SA2          ************************************************************
SA3          ************************************************************
SA12         ************************************************************
SA16         ************************************************************
SA17         ************************************************************
SA18         ************************************************************
SA19         ************************************************************
SA20         ************************************************************
SA21         ************************************************************

ATCC25923    GTTAACAAAACACAAGTGAAAATGGCAGTTG
SA2          *******************************
SA3          *******************************
SA12         *******************************
SA16         *******************************
SA17         *******************************
SA18         *******************************
SA19         *******************************
SA20         *******************************
SA21         *******************************
```

L4 of *S. epidermidis*

```
ATCC12228      GAAGCGAAAGGAGGAAATTGCATAATGGCTAATTATGATGTTTTAAAAGTAGACGGATCA
SE7            ************************************************************
SE9            ************************************************************
SE10           ************************************************************

ATCC12228      AAATCAGGTTCAGTTGAATTAAACGATGCAGTATTTGCTATCGAACCTAATAATAGCGTT
SE7            ************************************************************
SE9            ************************************************************
SE10           ************************************************************

ATCC12228      CTTTTTGAAGCTATTAACTTACAACGTGCGTCATTACGCCAAGGTACTCACGCTGTTAAG
SE7            ************************************************************
SE9            ************************************************************
SE10           ************************************************************

ATCC12228      AATCGTTCAGCAGTACGTGGTGGTGGACGTAAACCATGGAGACAAAAAGGTACAGGTCGT
SE7            ************************************************************
SE9            ************************************************************
SE10           ************************************************************

ATCC12228      GCACGTCAAGGTACAATCCGTGCTCCACAATGGCGTGGTGGTGGTGTAGTATTCGGACCA
SE7            ************************************************************
SE9            ************************************************************
SE10           ************************************************************

ATCC12228      ACACCAAGAAGCTATGCATATAAAATGCCAAAGAAAATGCGTCGTTTAGCATTACGTTCT
SE7            ************************************************************
SE9            ************************************************************
SE10           ************************************************************

ATCC12228      GCATTATCTTTTAAAGTTCAAGAAAATAGCTTTACAATTGTAGATACTTTTGGTTTTGAA
SE7            ************************************************************
SE9            ************************************************************
SE10           ************************************************************

ATCC12228      GCACCAAAAACAAAAGAATTCAAAAATGTATTAACTACTCTTGAACAACCTAAGAAAGTA
SE7            ************************************************************
SE9            ************************************************************
SE10           ************************************************************

ATCC12228      TTAGTTGTAACAGAAAACGAAGATGTAAATGTTGAATTATCAGCACGTAACATTCCTGGT
SE7            ************************************************************
SE9            ************************************************************
SE10           ************************************************************

ATCC12228      GTTCAAGTTACAACTGCTCAAGGATTAAATGTACTTGATCTAACAAGCGCTGACAGTGTA
SE7            ************************************************************
SE9            ************************************************************
SE10           ************************************************************

ATCC12228      ATCATTACAGAAGCAGCTGCGAAAAAAGTTGAGGAGGTGCTCGCATAATGGAAGCAAGAG
SE7            ************************************************************
SE9            ************************************************************
SE10           ************************************************************

ATCC12228      ATGTTCTTAAGCGCCCCGTAATCACTGAAAAATCTTCTGAAGCTATGGCAGAAGATAAAT
SE7            ************************************************************
SE9            ************************************************************
SE10           ************************************************************

ATCC12228      ACACTTTCGATGTAGATACTCGTGCAAATAAAACACAAGTTAAAATTGCTGTTGAAGAAA
SE7            ************************************************************
SE9            ************************************************************
SE10           ************************************************************
```

ATCC12228	TCTTCGA
SE7	*******
SE9	*******
SE10	*******

L4 of *S. sciuri*

```
NCTC12103      GGTAACGTGCCAGGTCCTAAAAAAGGTTTAGTAAAACTTACAACTTCAATTAAAAAAGGT
SSC1           ************************************************************
SSC2           ************************************************************

NCTC12103      AATAAATAATTATTTAGGTGAAAGGAGGAAACAACATTATGGCTAATATTGATGTATTAA
SSC1           ************************************************************
SSC2           ************************************************************

NCTC12103      AAGTAGATGGATCAAAATCAGGTTCTATCGAATTAAATGATAGTGTATTTGGTATCGAAC
SSC1           ************************************************************
SSC2           ************************************************************

NCTC12103      CAAACCAACACGTATTATTCGAAGCAGTCAACTTACAACGTGCTTCATTACGCCAAGGTA
SSC1           ************************************************************
SSC2           ************************************************************

NCTC12103      CTCACGCAGTTAAAAATCGTTCAGCAGTTCGTGGTGGTGGACGTAAACCTTGGAAACAAA
SSC1           ************************************************************
SSC2           ************************************************************

NCTC12103      AAGGTACAGGTCGTGCTCGTCAAGGTACAATCCGTGCGCCACAATGGCGTGGTGGTGGTA
SSC1           ************************************************************
SSC2           ************************************************************

NCTC12103      TCGTATTCGGTCCTACACCAAGAAGTTATTCTTACAAAATGCCTAAGAAAATGCGTCGTT
SSC1           ************************************************************
SSC2           ************************************************************

NCTC12103      TAGCATTACGTTCTGCATTATCTTCAAAAGTAAGTGGTAACGAATTAACAGTTTTAGAAG
SSC1           ************************************************************
SSC2           ************************************************************

NCTC12103      CATTCAACTTTGATGCACCTAAAACAAAAGAATTCAAAAATGTAATGAGCAACTTAGAAC
SSC1           ************************************************************
SSC2           ************************************************************

NCTC12103      TTTCTAAGAAAGTTTTATTCATCGTTGATACTGTAGACAGCAACGTTGAATTATCAGCTC
SSC1           ************************************************************
SSC2           ************************************************************

NCTC12103      GTAACATTCCAGGTGTTAAAGTTATCGACGCTCAAAGCTTAAACGTATTAGATATCTTAG
SSC1           ************************************************************
SSC2           ************************************************************

NCTC12103      ATTCTAGTAAAGTCGTAATAACTAAAGCAGCAGTGGATAAAGTAGAGGAGGTGCTCGCAT
SSC1           ************************************************************
SSC2           ************************************************************

NCTC12103      AATGGAAGCAAGAGATGTTATTAAGCGCCCCGTAATCACAGAAAAATCTTCATCAGATAT
SSC1           ************************************************************
SSC2           ************************************************************

NCTC12103      GGCTTTAGATAAATACACATTTGATGTAGATACACGTGCAAACAAAACACAAGTAAAAAT
SSC1           ************************************************************
SSC2           ************************************************************

NCTC12103      CGCAATCGAAGAAATTTTC
SSC1           *******************
SSC2           *******************
```

L4 of *S. simulans*

```
ATCC11046    AAAGGTAATGTGCCTGGTCCTAAAAAAGGATTAGTAGAAATCAAAACTTCAATTAAAAAA
SSM1         ************************************************************

ATCC11046    GGTAACAAATAATTAATTAACGAAAGGAGGAAAATGCATAATGGCAAATTATGATGTATT
SSM1         ************************************************************

ATCC11046    AAAAGTAGACGGATCAAAAGCAGGTTCAGTTGAATTAAGCGATGCTGTATTCGCAATTGA
SSM1         ************************************************************

ATCC11046    ACCAAACAAAGATGTTCTTTTCGAAGCAATTAACTTACAACGTGCTTCATTACGCCAAGG
SSM1         ************************************************************

ATCC11046    TACGCATTCGGTAAAAAACCGTTCAGCAGTACGCGGCGGTGGACGTAAACCATGGAGACA
SSM1         ************************************************************

ATCC11046    AAAAGGTACAGGACGTGCGCGTCAAGGTACTATCCGTGCTCCACAATGGCGTGGTGGCGG
SSM1         ************************************************************

ATCC11046    TATCGTATTCGGACCGACTCCAAGAAGCTATGCATACAAAATGCCTAAGAAAATGCGTCG
SSM1         ************************************************************

ATCC11046    TTTAGCATTACGTTCTGCATTATCTTTCAAAGTACAAGAAAATGAATTCAAAATTATTGA
SSM1         ************************************************************

ATCC11046    TGCTTTCGGTTTAGAAGCACCAAAAACTAAAGAATTCACTAAAGTATTATCAAACTTAGA
SSM1         ************************************************************

ATCC11046    ATTACCTAAAAAAGTTTTAGTAGTTACTGAATCAGAAGATGTAAATGTTGAATTATCAGC
SSM1         ************************************************************

ATCC11046    ACGTAACATTCCTGGTGTTCAAATCACTACTGTAACTGGTTTAAACGTATTAGACATCAC
SSM1         ************************************************************

ATCC11046    TAGCGCTGACAGTGTATTAATTACAGAAGCAGCTGCTAAAAAAGTTGAGGAGGTGCTCGG
SSM1         ************************************************************

ATCC11046    ATAATGGAAGCAAGAGACGTTCTTAAGCGCCCCGTAATCACAGAAAAATCTTCAGAAGCA
SSM1         ************************************************************

ATCC11046    ATGGCTGAAGACAAATACACTTTTGATGTAGATACACGTGCGA
SSM1         *******************************************
```

L22 of *S. aureus*

```
ATCC25923     GCTCAAGAAGGAAGCGAAAAGAAACAAGTAATCAAAACATGGTCACGTCGTTCTACAATT
SA2           ************************************************************
SA3           ************************************************************
SA12          ************************************************************
SA16          ************************************************************
SA17          ************************************************************
SA18          ************************************************************
SA19          ************************************************************
SA20          ************************************************************
SA21          ************************************************************

ATCC25923     TTCCCTAATTTCATCGGACATACTTTTGCAGTATACGACGGACGTAAACACGTACCTGTA
SA2           ************************************************************
SA3           ************************************************************
SA12          ************************************************************
SA16          ************************************************************
SA17          ************************************************************
SA18          ************************************************************
SA19          ************************************************************
SA20          ************************************************************
SA21          ************************************************************

ATCC25923     TATGTAACTGAAGATATGGTAGGTCATAAATTAGGTGAGTTTGCTCCTACTCGTACATTC
SA2           ************************************************************
SA3           ************************************************************
SA12          ************************************************************
SA16          ************************************************************
SA17          ************************************************************
SA18          ************************************************************
SA19          ************************************************************
SA20          ************************************************************
SA21          ************************************************************

ATCC25923     AAAGGACACGTTGCAGACGACAAGAAAACAAGAAGATAATATCTATTAAGTAGAGGAGGA
SA2           ************************************************************
SA3           ************************************************************
SA12          ************************************************************
SA16          ************************************************************
SA17          ************************************************************
SA18          ************************************************************
SA19          ************************************************************
SA20          ************************************************************
SA21          ************************************************************

ATCC25923     CATCCTAATGGAAGCAAAAGCGGTTGCTAGAACAATAAGAATCGCACCTCGTAAAGTAAG
SA2           ************************************************************
SA3           ************************************************************
SA12          ************************************************************
SA16          ************************************************************
SA17          ************************************************************
SA18          ************************************************************
SA19          ************************************************************
SA20          ************************************************************
SA21          ************************************************************


ATCC25923     ACTAGTTCTTGACTTAATCAGAGGTAAAAATGCTGCTGAAGCTATTGCAATTTTAAAATT
SA2           ************************************************************
SA3           ************************************************************
SA12          ************************************************************
SA16          ************************************************************
SA17          ************************************************************
SA18          ************************************************************
SA19          ************************************************************
```

```
SA20           ************************************************************
SA21           ************************************************************

ATCC25923      AACAAACAAAGCTTCATCACCAGTAATTGAAAAAGTATTAATGTCCGCTTTAGCTAATGC
SA2            ************************************************************
SA3            ************************************************************
SA12           ************************************************************
SA16           ************************************************************
SA17           ************************************************************
SA18           ************************************************************
SA19           ************************************************************
SA20           ************************************************************
SA21           ************************************************************

ATCC25923      TGAACATAACTATGACATGAACACAGATGAATTAGTAGTTAAAGAAGCATATGCTAACGA
SA2            ************************************************************
SA3            ************************************************************
SA12           ************************************************************
SA16           ************************************************************
SA17           ************************************************************
SA18           ************************************************************
SA19           ************************************************************
SA20           ************************************************************
SA21           ************************************************************

ATCC25923      AGGACCAACATTAAAACGTTTCCGTCCACGTGCGCAAGGTCGTGCAAGTGCGATTAACAA
SA2            ************************************************************
SA3            ************************************************************
SA12           ************************************************************
SA16           ************************************************************
SA17           ************************************************************
SA18           ************************************************************
SA19           ************************************************************
SA20           ************************************************************
SA21           ************************************************************

ATCC25923      ACGTACAAGCCACATTACAATCGTCGTAAGTGACGGTAAAGAAGAAGCTAAAGAAGCTTA
SA2            ************************************************************
SA3            ************************************************************
SA12           ************************************************************
SA16           ************************************************************
SA17           ************************************************************
SA18           ************************************************************
SA19           ************************************************************
SA20           ************************************************************
SA21           ************************************************************

ATCC25923      ATTAACTTTTAAGGAGGGAATACTGTGGGTCAAAAAATTAATCCAATCGGACTTCGTGTT
SA2            ************************************************************
SA3            ************************************************************
SA12           ************************************************************
SA16           ************************************************************
SA17           ************************************************************
SA18           ************************************************************
SA19           ************************************************************
SA20           ************************************************************
SA21           ************************************************************

ATCC25923      GGTATTATCCGTGATTGGGAAGCTAAATGGTATGCTGAAAAAGACTTCGCTTCACTTTTA
SA2            ************************************************************
SA3            ************************************************************
SA12           ************************************************************
SA16           ************************************************************
SA17           ************************************************************
SA18           ************************************************************
SA19           ************************************************************
SA20           ************************************************************
```

```
SA21         ************************************************************

ATCC25923    CACGAAGATTTAAAAATCCGTAAATTTATTGATAATGAATTAAAAGAAGCATCAGTTTCT
SA2          ************************************************************
SA3          ************************************************************
SA12         ************************************************************
SA16         ************************************************************
SA17         ************************************************************
SA18         ************************************************************
SA19         ************************************************************
SA20         ************************************************************
SA21         ************************************************************

ATCC25923    CACGTAGAGATTGAACGTGCTGCAAACCGTATCAACATTGCAATTCATACTGGTAAACCT
SA2          ************************************************************
SA3          ************************************************************
SA12         ************************************************************
SA16         ************************************************************
SA17         ************************************************************
SA18         ************************************************************
SA19         ************************************************************
SA20         ************************************************************
SA21         ************************************************************

ATCC25923    GGTATGGTAATTGGTAAAGGCGGTTCAGAAATCGAAAAATTACGCAACAAATTAAATGCG
SA2          ************************************************************
SA3          ************************************************************
SA12         ************************************************************
SA16         ************************************************************
SA17         ************************************************************
SA18         ************************************************************
SA19         ************************************************************
SA20         ************************************************************
SA21         ************************************************************
```

L22 of *S. epidermidis*

```
ATCC12228    CATTGGCCATACTTTTGCAGTATACGATGGACGTAAACATGTACCTGTATATGTCACTGA
SE7          ************************************************************
SE9          ************************************************************
SE10         ************************************************************

ATCC12228    AGATATGGTAGGTCACAAATTAGGTGAATTTGCTCCTACACGTACTTTTAAAGGACATGC
SE7          ************************************************************
SE9          ************************************************************
SE10         ************************************************************

ATCC12228    AGCAGACGACAAGAAAACAAGAAGATAATTTTAATAAGTAGAGGAGGAAATCCTGATGGA
SE7          ************************************************************
SE9          ************************************************************
SE10         ************************************************************

ATCC12228    AGCAAAAGCGGTTGCTAGAACAATAAGAATCGCACCTCGTAAAGTAAGACTTGTTCTTGA
SE7          ************************************************************
SE9          ************************************************************
SE10         ************************************************************

ATCC12228    TTTAATCAGAGGTAAAAATGCTGGAGAAGCTATTGCTATTTTAAAATTAACTAATAAAGC
SE7          ************************************************************
SE9          ************************************************************
SE10         ************************************************************

ATCC12228    TTCATCACCAGTAATCGAAAAAGTATTAATGTCCGCTTTGGCAAATGCTGAGCACAACTA
SE7          ************************************************************
SE9          ************************************************************
SE10         ************************************************************

ATCC12228    TGATATGAATACAGATGAATTAGTTGTAAAAGAAGCATATGCTAATGAAGGACCAACTTT
SE7          ************************************************************
SE9          ************************************************************
SE10         ************************************************************

ATCC12228    AAAACGTTTCCGCCCACGTGCACAAGGACGTGCAAGTGCAATTAATAAACGTACAAGCCA
SE7          ************************************************************
SE9          ************************************************************
SE10         ************************************************************

ATCC12228    CATTACAATCGTCGTAAGTGACGGTAAAGAAGAAGCTAAAGAAGCTTAATAACTTTCTAA
SE7          ************************************************************
SE9          ************************************************************
SE10         ************************************************************

ATCC12228    GGAGGGAATACTGTGGGTCAAAAAATTAATCCAATCGGACTTCGTGTCGGTGTTATCCGT
SE7          ************************************************************
SE9          ************************************************************
SE10         ************************************************************

ATCC12228    GATTGGGAAGCAAAATGGTATGCTGAAAAAGACTTTGCTTCACTTTTACATGAAGACTTA
SE7          ************************************************************
SE9          ************************************************************
SE10         ************************************************************

ATCC12228    AAAATCCGTAAATTTATTGATAATGAATTAAAAGAAGCGTCAGTTTCTCACGTAGATATT
SE7          ************************************************************
SE9          ************************************************************
SE10         ************************************************************

ATCC12228    GAACGTGCAGCAAATCGCATCAATATTGC
SE7          *****************************
SE9          *****************************
SE10         *****************************
```

L22 of *S. sciuri*

```
NCTC12103     TGGTCTCGTCGTTCAACAGTATTTCCTAACTTCATCGGACATACAATAGCAGTATATGAT
SSC1          ************************************************************
SSC2          ************************************************************

NCTC12103     GGTCGTAAACATGTGCCAGTTTTCATTACTGAAGATATGGTTGGACATAAATTAGGAGAG
SSC1          ************************************************************
SSC2          ************************************************************

NCTC12103     TTTGCACCTACACGTACTTATAAAGGTCATGCTGCAGACGACAAAAAGACTAAACGCTAA
SSC1          ************************************************************
SSC2          ************************************************************

NCTC12103     TTTCTAAAAAGAGGAGGAAGTCACAATGGAAGCAAAAGCGGTTGCTAGAACAATAAGAAT
SSC1          ************************************************************
SSC2          ************************************************************

NCTC12103     CGCACCTCGTAAAGTGAGATTAGTTTTAGATCTTATTAGAGGTAAAGAAGTGGCAGAAGC
SSC1          ************************************************************
SSC2          ************************************************************

NCTC12103     AATTGCCATCTTAAAATTAACAAACAAAGCAACATCACCTGTTGTTGAAAAATTATTAAT
SSC1          ************************************************************
SSC2          ************************************************************

NCTC12103     GTCAGCATTAGCAAATGCAGAACATAACTATGATTTAAATCCTGACACATTAGTAGTAAA
SSC1          ************************************************************
SSC2          ************************************************************

NCTC12103     AGAAGCTTACGCTAATGAAGGACCTACATTGAAACGTTTCCGTCCACGTGCACAAGGTCG
SSC1          ************************************************************
SSC2          ************************************************************

NCTC12103     TGCAAGTGCGATTAACAAACGTACTAGCCATATTACAATCGTAGTTGGCGAAAAAGAAGC
SSC1          ************************************************************
SSC2          ************************************************************

NCTC12103     TAAAGAAGTTAAAGAAGCATAATTAACTAAATCATTAAGGAGGGAATATAGTGGGTCAAA
SSC1          ************************************************************
SSC2          ************************************************************

NCTC12103     AAATTAATCCAATCGGACTTCGTGTCGGTGTAATTCGTGACTGGGAAGCAAAGTGGTATG
SSC1          ************************************************************
SSC2          ************************************************************

NCTC12103     CAGGTAAAGACTTCGCAACACTTTTACATGAAGACTTAAAAGTTCGTAAATATATCGCAG
SSC1          ************************************************************
SSC2          ************************************************************

NCTC12103     AAGCATTAAAAGAAGCTTCAGTTTCAAGTGTTGAAATTGAACGTGCAGCAAA
SSC1          ****************************************************
SSC2          ****************************************************
```

L22 of *S. simulans*

```
ATCC11046     GACATGCTGCAGACGACAAAAAAACAAGAAGATAAATACTTTCTATTAAGTAGAGGAGGA
SSM1          ************************************************************

ATCC11046     AGACCAAATGGAAGCAAAAGCGGTTGCTAGAACTATCAGAATCGCACCTCGTAAAGTCAG
SSM1          ************************************************************

ATCC11046     ATTAGTTTTAGACTTAATCAGAGGCAAAAACGTTGGTGAAGCTGTTGCTATTTTAAAATT
SSM1          ************************************************************

ATCC11046     AACTAACAAAGCATCATCACCAGTTGTTGAAAAATTATTAATGTCCGCTTTAGCTAATGC
SSM1          ************************************************************

ATCC11046     AGAGCACAACTATGACATGAATACAGATGAATTAGTTGTTAAAGAAGCATATGCTAACGA
SSM1          ************************************************************

ATCC11046     AGGACCAACTTTAAAACGTTTCCGTCCACGTGCTCAAGGACGCGCAAGTGCAATTAACAA
SSM1          ************************************************************

ATCC11046     ACGTACAAGCCACATTACAATTGTCGTAAGTGACGGCAAAGAAGAAGCTAAAGAAGCTTA
SSM1          ************************************************************

ATCC11046     ATAAACTATCTAAGGAGGGAATACTGTGGGTCAAAAAATTAATCCAATCGGACTTCGTGT
SSM1          ************************************************************

ATCC11046     TGGTGTTATCCGTGATTGGGACGCTAAATGG
SSM1          *******************************
```
